# Antioxidant and Phytochemical Potential of and Phytochemicals in *Gymnema inodorum* (Lour.) Decne in Northern Thailand

**DOI:** 10.3390/plants11243498

**Published:** 2022-12-13

**Authors:** Natwasan Jeytawan, Sumed Yadoung, Peerapong Jeeno, Pichamon Yana, Kunrunya Sutan, Warangkana Naksen, Malaiporn Wongkaew, Sarana Rose Sommano, Surat Hongsibsong

**Affiliations:** 1 Faculty of Public Health, Chiang Mai University, Chiang Mai 50200, Thailand; 2 Environmental, Occupational Health Sciences Research and NCD Center of Excellence, Research Institute for Health Sciences, Chiang Mai University, Chiang Mai 50200, Thailand; 3 Environmental Science Program, Faculty of Science, Chiang Mai University, Chiang Mai 50200, Thailand; 4 School of Health Science Research, Research Institute for Health Sciences, Chiang Mai University, Chiang Mai 50200, Thailand; 5 Program of Food Production and Innovation, Faculty of Integrated Science and Technology, Rajamangala University of Technology Lanna, Chiang Mai 50300, Thailand; 6 Plant Bioactive Compound Laboratory, Faculty of Agriculture, Chiang Mai University, Chiang Mai 50200, Thailand

**Keywords:** *Gymnema inodorum* (Lour.) Decne, antioxidant, phytochemical, gymnemic acid, LC-QTOF/MS

## Abstract

*Gymnema inodorum* (Lour.) Decne is a vegetable local to Chiang Mai Province of Northern Thailand. This study aimed to analyze the antioxidant and phytochemical potential of *G. inodorum* found in Chiang Mai Province; antioxidant compounds of *G. inodorum* were tested via DPPH, ABTS and FRAP assays, and total phenolic compound and total flavonoid contents were analyzed. Anti-inflammatory effects were focused on regarding pharmacological potential. The gymnemic acid level was analyzed by HPLC-UV, and other potential chemicals were analyzed by LC-QTOF/MS. The quantifications of gymnemic acid contents analyzed using HPLC-UV showed that the highest gymnemic acid concentrations were found in the air-dried and roasted 1-day-fermented leaf extracts (0.1258 ± 0.0157 μg/mg). The highest free radical scavenging activity via DPPH assay was found in baked leaf extract, with an IC_50_ of 8.99 mg/mL, and via ABTS assay in baked and roasted leaf extracts, with an IC_50_ of 1.05 mg/mL. FRAP assays showed the highest free radical scavenging activity for the baked leaf extract, with 0.0085 ± 0.008 mM Fe^2+^/g sample. The total phenolic contents of fresh *G. inodorum* leaf extracts obtained with ethanol, methanol and water were 0.19 ± 0.0004, 0.21 ± 0.0010 and 0.10 ± 0.0008 μg GAE/g, respectively. The total flavonoid contents of fresh *G. inodorum* leaf extracts obtained with ethanol, methanol and water were 74.56 ± 28.00, 71.88 ± 16.11 and 10.74 ± 3.63 μg QE/g, respectively. The LC-QTOF/MS analysis of the fresh *G. inodorum* leaf extract showed that 6-hydroxykaempferol 7-rutinoside was the most abundant compound. In the study, *G. inodorum*, a plant local to Northern Thailand, is shown to be a useful plant with high antioxidant and phytochemical potential properties.

## 1. Introduction

Gymnema is a genus in the Apocynaceae family, commonly known as the dogbane family. Currently, there are more than 50 species of Gymnema that can be found around the world, including annual plants, perennial herbs, stem succulents, woody shrubs, trees and vines [1,2,3]. When they are cut, they mostly exude a milky latex [4]. The general characteristics of this genus have been described since 1810: the leaves are uncomplicated, appear singly on alternating sides of the stem (alternate) [2] and are usually found in pairs and rarely in circles, such that the pairs occur on opposite sides of the stem by a 90° rotation of the lower leaf [1] and have no stipule (a small, leaf-like structure at the base of the leaf stem) or small stipules that are probably fingerlike [2]. The flowers are bisexual, actinomorphic (radial symmetry) and borne in heads that can be cymes, racemes or solitary in axils, with a synsepalous, five-lobed calyx united into a tube at the base [1,5].

*Gymnema sylvestre*, one frequent species, is commonly used as a dietary supplementary food for its ability to suppress the taste of sweetness [6,7]. *G. inodorum* is a native plant that is found in some countries of South China and India and is mostly found on the Southeast Asian continent, especially in Thailand [8]. This plant is characterized by broad lanceolate leaves with smooth edges that are slender and pointed at the tip and curved at the base, with green stems that become brown at maturity. Normally, *G. inodorum* can be found in evergreen and deciduous forests, which are most frequently found in tropical forests [9]. Currently, in Thailand, *G. inodorum*, called Chiang Da, is usually planted for cooking, being the main ingredient in many dishes, boiled and stir-fried, and is also air-dried to make tea, especially in the countryside.

Reportedly, *G. sylvestre* (a closely related species of *G. inodorum*) contains an abundance of phytoconstituents responsible for sweet-suppression activity, including triterpene saponins, known as gymnemic acids and gymnemasaponins, and a polypeptide, gurmarin [10]. Gymnemic acid is an important chemical compound of phytochemical groups that can be responsible for regulating sugar levels in the bloodstream [11,12]. Moreover, some previous studies found a high antioxidative ability [13]. The department of Thai Traditional and Alternative Medicine states that *G. inodorum* is a herb; it can decrease blood sugar levels and provide a variety of nutrients, beta-carotene and multivitamins, which are known as antioxidant substances [14]. However, there have been many studies on Gymnema for chemical compounds and pharmaceutical properties, which have been mostly conducted on *G. sylvestre*; this is more widespread in many countries, such as South China, the Ryukyu Islands, India, Sri Lanka and the continent of Africa and Southeast Asia [3]. Therefore, the study of *G. inodorum*, which is frequently found in this region, is very important, especially for people who eat this species of plant, such as people in Northern Thailand.

The aim of the present study was to analyze the antioxidants and phytochemical potential in *G. inodorum* from Chiang Mai Province, Northern Thailand. The analysis was focused on antioxidants analyzed by DPPH, ABTS and FRAP assays, total phenolic compounds, total flavonoid contents and pharmacological potential, with attention being paid to anti-inflammatory and gymnemic acid levels.

## 2. Results

**Figure 1:**
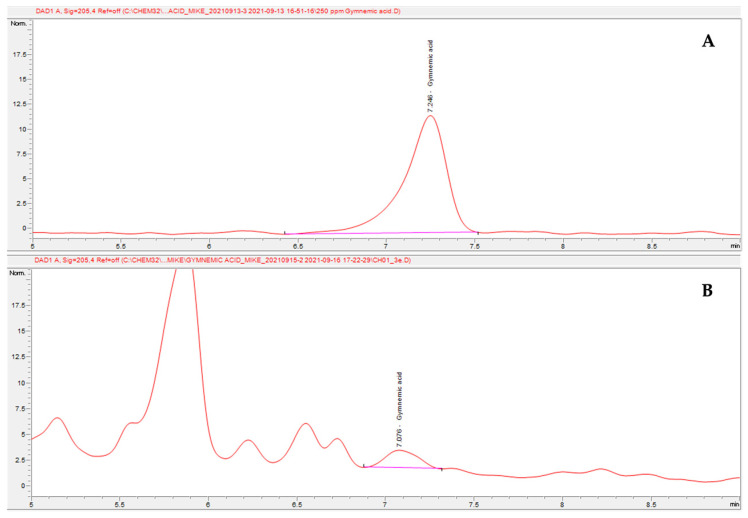
Chromatogram of gymnemic acid from HPLC-UV (210 nm): (**A**) standard gymnemic acid at 1 ppm; (**B**) gymnemic acid in the sample.

Gymnemic acid was obtained from *G. inodorum* samples. Based on the results, the highest gymnemic acid concentrations were found in air-dried and roasted 1-day-fermented leaves at 0.1258 ± 0.0157 µg/mg, followed by baked leaves (0.1020 ± 0.0122 µg/mg), air-dried and roasted leaves (0.0974 ± 0.0115 µg/mg), baked and roasted leaves (0.0786 ± 0.0086 µg/mg), air-dried leaves (0.0642 ± 0.0064 µg/mg), air-dried and roasted 30 s-boiled leaves (0.0578 ± 0.0066 µg/mg) and air-dried and roasted 60 s-boiled leaves (0.0460 ± 0.0037 µg/mg), with the lowest concentrations being found in fresh leaves (0.0194 ± 0.0001 µg/mg). The results showed the lowest concentrations of gymnemic acid in fresh leaves and the highest in air-dried and roasted 1-day-fermented leaves, followed by baked leaves (Table 1). An example chromatogram from HPLC-UV is shown in Figure 1.

**Figure 2:**
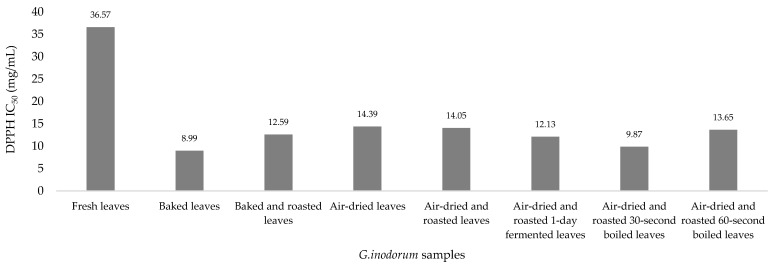
Bar graph depicting the DPPH radical scavenging activity of *G. inodorum* (Lour.) Decne.

The antioxidant activity in *G. inodorum* was determined with three different reagents, and the results was shown in Table 2. In the DPPH assay, the highest antioxidant activity was found in baked leaves (IC_50_: 8.99 mg/mL), followed by air-dried and roasted 30 s-boiled leaves (IC_50_: 9.87 mg/mL), air-dried and roasted 1-day-fermented leaves (IC_50_: 12.13 mg/mL), baked and roasted leaves (IC_50_: 12.59 mg/mL), air-dried and roasted 60 s-boiled leaves (IC_50_: 13.65 mg/mL), air-dried and roasted leaves (IC_50_: 14.05 mg/mL) and air-dried leaves (IC_50_: 14.39 mg/mL), and the lowest antioxidant activity was found in fresh leaves (IC_50_: 36.57 mg/mL). There were significantly higher DPPH assay results for air-dried and roasted 30 s-boiled leaves and air-dried and roasted 60 s-boiled leaves than for fresh leaves (*p* = 0.014), while the others showed no significant differences (Figure 2).

Similarly, the ABTS reagent assay showed the highest antioxidant activity in baked and roasted leaves (IC_50_: 1.05 mg/mL), followed by baked leaves (IC_50_: 1.11 mg/mL), air-dried leaves (IC_50_: 1.30 mg/mL), air-dried and roasted 60 s-boiled leaves (IC_50_: 4.05 mg/mL), air-dried and roasted 30 s-boiled leaves (IC_50_: 5.03 mg/mL) and fresh leaves (IC_50_: 12.79 mg/mL). However, air-dried and roasted leaves and air-dried and roasted 1-day-fermented leaves were not detectable with ABTS. Similarly, the ferric ion reducing antioxidant power results obtained by FRAP assays revealed the highest antioxidant activity in baked leaves (0.0085 ± 0.008 mM Fe^2+^/g), followed by air-dried leaves (0.0075 ± 0.008 mM Fe^2+^/g), baked and roasted leaves (0.0070 ± 0.008 mM Fe^2+^/g), air-dried and roasted 1-day-fermented leaves (0.0070 ± 0.007 mM Fe^2+^/g), air-dried and roasted 30 s-boiled leaves (0.0064 ± 0.007 mM Fe^2+^/g), air-dried and roasted leaves (0.0059 ± 0.007 mM Fe^2+^/g) and air-dried and roasted 60 s-boiled leaves (0.0055 ± 0.006 mM Fe^2+^/g), with the lowest in fresh leaves (0.0013 ± 0.002 mM Fe^2+^/g). However, there were no significant differences in the ABTS and FRAP assay results for *G. inodorum* (*p* < 0.05).

The phenolic content was analyzed in *G. inodorum* leaves. The total phenolic content of fresh *G. inodorum* leaves showed that methanol extract had the highest phenolic content at 0.21 ± 0.0010 µg GAE/g, followed by ethanol and H₂O extracts at 0.19 ± 0.0004 µg GAE/g and 0.10 ± 0.0008 µg GAE/g, respectively (Table 3).

The total flavonoid content of fresh *G. inodorum* leaves showed that ethanol extract had the highest flavonoid content at 74.56 ± 28.00 µg QE/g, followed by methanol and H₂O extracts at 71.88 ± 16.11 µg QE/g and 10.74 ± 3.63 µg QE/g, respectively (Table 3).

The anti-inflammatory properties of fresh leaves showed 82.79 ± 2.28% protection and 17.21 ± 2.28% hemolysis (Table 4).

**Figure 3:**
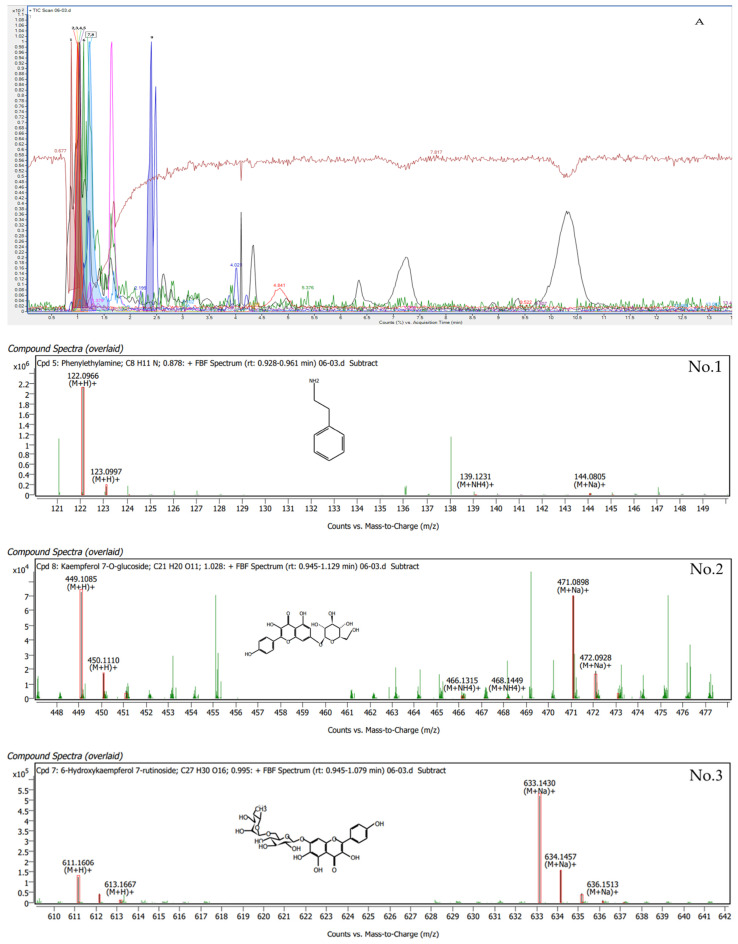

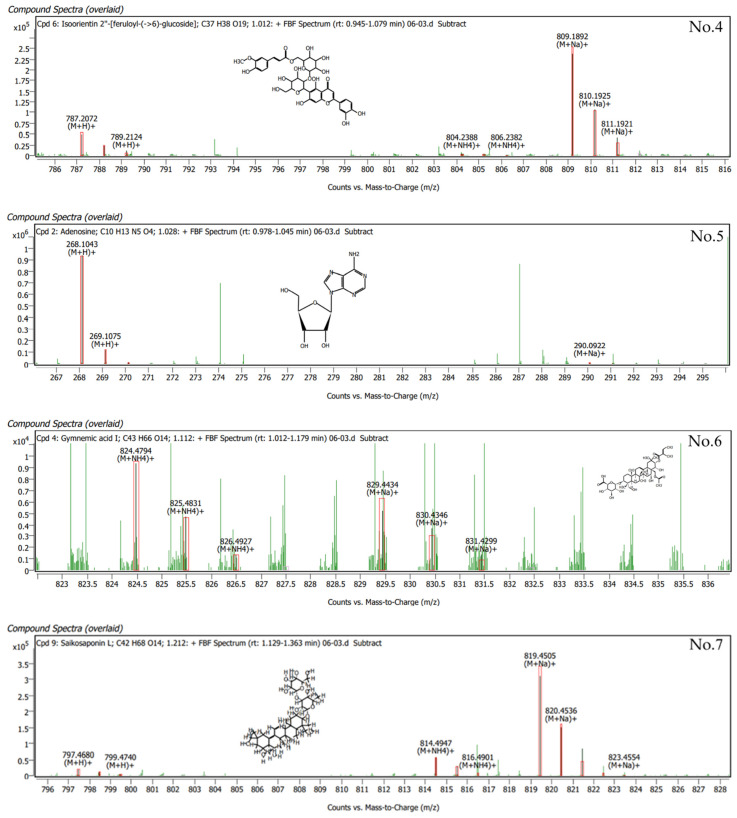

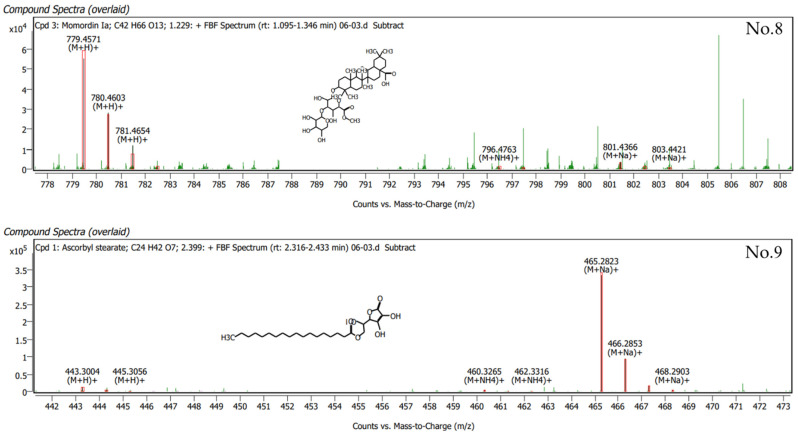
LC-QTOF/MS analysis of the top nine compounds in terms of retention times for *G. inodorum*.

Phytochemical compounds were found in *G. inodorum* leaves extracted by LC-QTOF/MS. The compounds in *G. inodorum* revealed by LC–QTOF/MS analysis are listed in Table 5, and the LC-QTOF/MS chromatograms are shown in Figure 3. Among these, all compounds produced a matching score >90% and were identified as 6-hydroxykaempferol 7-rutinoside, ascorbyl stearate, adenosine, gymnemic acid I, phenethylamine, momordin Ia, saikosaponin L, isoorientin 2″-[feruloyl-(->6)-glucoside] and kaempferol 7-O-glucoside. The highest matching score (99.41%) obtained for the G. inodorum leaf extracts was that for 6-hydroxykaempferol 7-rutinoside. 6-Hydroxykaempferol 7-rutinoside is an antioxidant flavonoid that helps to suppress and slow down the free-radical-causing oxidative stress process, which stimulates the body’s inflammatory response. Gymnemic acid I (90.05%) is a class of chemical compounds isolated from the leaves of *G. inodorum*. They are anti-sweet compounds or sweetness inhibitors.

## 3. Discussion

In this investigation, different methods for determining the gymnemic acid contents in *G. inodorum* were studied using different solvents. Our results suggested that air-dried and roasted 1-day-fermented *G. inodorum* leaves were best for obtaining the most gymnemic acid. Interestingly, fermentation may help the degradation of plant leaf structures, facilitating the extraction of more biochemical components.

Based on the results, the amount of gymnemic acid found in this study was 19.4 ± 0.0001 µg/g of fresh leaves. When compared with previous studies, the amounts of gymnemic acid extracted in this study were lower than those obtained in a study from Chiang Mai Province, Thailand, in 2018 [15], and a study from Chiang Rai Province, Thailand, in 2019 [16], which were extracted from the same *G. inodorum*. Regardless of the methods of extraction, the gymnemic acid concentrations varied widely. However, samples obtained in different areas may contain different amounts of gymnemic acid [17].

The antioxidant capacity test revealed that the fresh leaves had a lower antioxidant capacity than the processed samples. Conversely, fresh fruits and vegetables are reported to contain more antioxidant activity, and this antioxidant capacity declines when moisture is lost and with high temperature [18]. Therefore, this lower antioxidant capacity might be due to the absence of dry and wet weight adjustment, which was one limitation of this study. However, from the antioxidant capacity test, the three assays showed similar results: the baked process yielded the highest antioxidant capacity, with the lowest values obtained in the DPPH and ABTS assays (with roasting) and the highest in the FRAP assay. This result was similar to a previous study that suggested that most antioxidants were found in dried samples of *G. inodorum* [19].

The difference in the extraction of phenolic and flavonoid contents revealed that methanol extraction provided the highest phenolic contents and that ethanol extraction provided the highest flavonoid contents. Our results suggested the appropriate solvents to obtain the highest phenolic and flavonoid contents from fresh *G. inodorum* leaves.

*G. inodorum* showed medical activity. The high protection percentage activity for anti-inflammation is useful with respect to developing *G. inodorum* leaves as future anti-inflammation drugs. However, this property still needs more study to elucidate the side effects and accurate medical properties.

## 4. Materials and Methods

### 4.1. Chemicals, Reagents and Equipment

The organic solvents methanol, acetonitrile and formic acid were obtained from J.T. Baker; ortho-phosphoric acid and dimethyl sulfoxide were obtained from ACI Labscan; and ethanol was obtained from ETHAL 95, Thailand. Chemicals: 1,1-Diphenyl-2-picryhydrazyl (DPPH), 2,4,6-tripyridyl-s-trizine (TPTZ) and 2,2-azino-bis(3-ethylbenzothiazoline-6-sulfonic acid) diammonium salt were purchased from Sigma–Aldrich; ferric chloride (FeCl3), sodium nitrite and potassium persulfate were purchased from LOBA CHEMIE PVT., LTD. Aluminum chloride was from QRëC, sodium hydroxide was from ACI Labscan, and Folin–Ciocalteu solution was purchased from Merck. Standards: Garlic acid (GAE) was purchased from Fluka, and rutin (RE) was purchased from SIGMA. Reagents: Alsever solution was obtained from SIGMA. Fetal bovine serum (FBS), Iscove’s modified Dulbecco’s medium (IMDM), 100,000 penicillin G (unit), trypsin solution and streptomycin sulfate (µg/mL) were purchased from GIBCO.

### 4.2. G. inodorum (Lour.) Decne Samples

**Figure 4:**
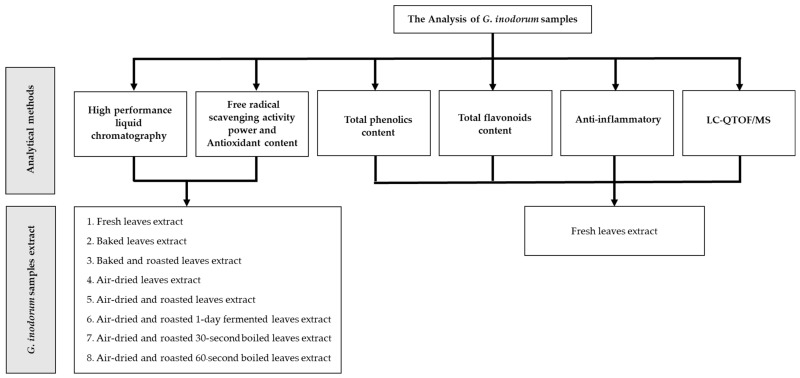
Flowchart of the methodology.

*G. inodorum* samples were obtained from Chiang Mai Province, Saraphi District, which is one of the most popular areas for *G. inodorum* commercial farms in Northern Thailand. The sample collection was conducted between January 2022 and February 2022, regardless of the season or time of collection. The samples collected included: 1. fresh leaves; 2. baked leaves prepared by baking in a hot air oven for 10–12 h; 3. air-dried leaves exposed to direct sunlight for 3–4 days, depending on the heat of the sun; 4. roasted leaves prepared using a roasting machine for approximately 45–60 min; and 5. fermented leaves that took 24 h to ferment. All samples were subsequently prepared by the appropriate method for each analysis performed in this study, as shown in Figure 4. All antioxidants, phytochemical functions and phytochemical compounds were analyzed in the laboratory of the Research Institute for Health Sciences (RIHES), Chiang Mai University, Thailand.

### 4.3. Gymnemic Acid Analysis

Eight testing samples were prepared for analysis by HPLC: 1. fresh leaves, 2. baked leaves, 3. baked and roasted leaves, 4. air-dried leaves, 5. air-dried and roasted leaves, 6. air-dried and roasted 1-day-fermented leaves, 7. air-dried and roasted 30 s-boiled leaves, and 8. leaves that were air-dried and roasted for 60 s. The samples were extracted by adding 3.0% potassium hydroxide in methanol for 1 h. The solution was dissolved in a mixture of methanol and DI water (1:1 *v*/*v*), followed by acidification with hydrochloric acid. The solution was adjusted with 50% methanol to 10 mL, filtered with nitrocellulose filter paper and kept at 20 °C until HPLC analysis.

The analysis consisted, briefly, of chromatographic measurements conducted using an Agilent Hewlett Packard (HP) 1100 HPLC System with a UV–visible absorbance detector. The C18 (Supelco) column was 25 cm × 4.6 mm, 5 µm and had a 205 nm wavelength. The mobile phases consisted of acetonitrile:0.1% ortho phosphoric acid (23:77 *v*/*v*), and there was a flow rate of 2.0 mL/min and an injection volume of 20 µL. All processes in the laboratory were maintained under ambient conditions [20].

### 4.4. Antioxidant Analysis

Eight testing samples were prepared to analyze the effect of *G. inodorum* treatment on free radical scavenging activity and antioxidant contents, including 1. fresh leaves, 2. baked leaves, 3. baked and roasted leaves, 4. air-dried leaves, 5. air-dried and roasted leaves, 6. air-dried and roasted 1-day-fermented leaves, 7. air-dried and roasted 30 s-boiled leaves, and 8. leaves that were air-dried and roasted for 60 s. The samples were extracted by boiling 2 g of *G. inodorum* in 100 mL of distilled water at 80 °C for 15 min, followed by cooling and filtration with filter paper No. 1. The free radical scavenging activities and antioxidant contents of the treated samples were compared with those of the raw samples.

#### 4.4.1. DPPH Radical Scavenging Activity

The free radical scavenging activity of the *G. inodorum* extracts were determined and modified by a 1,1-diphenyl-2-picryhydrazyl (DPPH) assay [21,22]. In brief, the DPPH stock solution was prepared by dissolving DPPH in methanol. One hundred microliters of DPPH working solution was mixed with 100 µL of plant extract solution in a 96-well plate and incubated in the dark for 30 min. The absorbance was measured at 517 nm using a microtiter plate reader. The DPPH free radical scavenging activity was calculated as a percentage using the following formula:

DPPH = [(Acontrol − Asample)/Acontrol] × 100 (1)

where Acontrol and Asample are the absorbance readings of the control and sample, respectively. The IC_50_ value for DPPH free radical scavenging activity corresponds to the sample concentration necessary to inhibit 50% of DPPH free radicals. The IC_50_ was determined graphically from the curve plot between the percentages of DPPH scavenging activity and the sample concentration.

#### 4.4.2. Ferric Ion Reducing Antioxidant Power (FRAP)

FRAP was determined by the modified Aljadai method [21,22]. In brief, the FRAP reagent was prepared by mixing 2.5 mL of 10 mM 2,4,6-tripyridyl-s-trizine (TPTZ) with 40 mM HCl, 2.5 mL of 20 mM ferric chloride (FeCl_3_) and 25 mL of 300 mM acetate buffer at pH 3.6. One gram of plant extracted solution was diluted two times with distilled water, and 10 µL of the extracted solution was mixed with 190 µL of FRAP reagent on a 96-well microtiter plate for 30 min in the dark. The sample’s absorbance was measured with a microtiter plate reader at 593 nm using ascorbic acid as a standard reference. FRAP was calculated using Equation (2) and reported as mg of ascorbic acid equivalent per 100 g of plant extract (mg AAE/100 g).

(2) $$\mathrm{AEAC}\;\left(\mathrm{mg}\;\mathrm{AAE}/100\;g\right)=\frac{C_{\mathrm{AA}\;}\times \;V\;\times \;\mathrm{DF}\;\times \;100}{\mathrm{weight}\;\mathrm{of}\;\mathrm{crude}\;\mathrm{extract}\;(g)}$$

where C_AA_ is the concentration of ascorbic acid from the standard curve (mg/mL), V is the volume of extract solution (mL) and DF is the dilution factor.

#### 4.4.3. ABTS Radical Scavenging Activity

To determine the antioxidative potential, ABTS was used, following the Arnao method [22,23]. In brief, 7 mM ABTS reagent was prepared using 2,2-azino-bis(3-ethylbenzothiazoline-6-sulfonic acid) diammonium salt in water and 2.45 mM of potassium persulfate incubated for 12 h in the dark at room temperature. The solution was diluted with 80% ethanol and then added to a 96-well plate containing 10 µL of plant extract. After incubation in the dark for 10 min, the absorbance was read at a wavelength of 734 nm and reported as IC50, which was the relationship between the percent inhibition of ABTS and the sample concentration. The percent inhibition of absorbance at 734 nm was calculated using Equation (3):

(3) $$\mathrm{ABTS}\;\mathrm{radical}\;\mathrm{scavenging}\;\mathrm{effect}\;(\%)=\left(\frac{A_{b\;}-{\;A}_{a}}{A_{b}}\right)\times 100$$

where A_b_ is the absorbance of ABTS radical + methanol and A_a_ is the absorbance of ABTS radical + sample extract/standard. Trolox was used as a standard substance.

### 4.5. Phytochemical Potential Analysis

#### 4.5.1. Extraction of Plant Samples for the Determination of Total Phenolic and Flavonoid Contents

Fresh leaves were cleaned and dried at 50 °C for 1 h and then crushed into small pieces. One gram of dried *G. inodorum* was added to 3 different solvents, water, ethanol and methanol, in quantities of 30 mL. Then, the samples were soaked for 3 days at room temperature, centrifuged at 2500 rpm for 5 min and filtered with a nitrocellulose filter. The resulting solution was dried with an evaporator. Finally, the extract was dissolved in dimethyl sulfoxide and kept at 4 °C until analysis [24].

#### 4.5.2. Total Phenolic Content

Total phenolic quantitative analysis was performed by modifying the method of Folin–Ciocalteu and Dewanto [25] using gallic acid (GAE) in 80% methanol as the standard reference and the results were reported in mg of gallic acid per gram of dry weight. The 12.5 µL sample was mixed with 12.5 µL of Folin–Ciocalteu solution (diluted 10 times in distilled water). Then, 125 µL of 7% sodium carbonate and 100 µL of distilled water were added. Absorption was then measured at 760 nm using a microplate reader spectrophotometer, and the total phenolic content was reported in mg garlic acid (GAE) per gram of dry weight.

#### 4.5.3. Total Flavonoid Content

The total flavonoid content was determined by colorimetric assay [26]. Briefly, 25 µL of sample solution was added to a 96-well plate with 7.5 µL of 7% sodium nitrite solution and 12.5 µL of distilled water. The solution was stored at room temperature for 5 min. Then, 15 µL of 10% aluminum chloride solution was added, mixed and kept at room temperature for 5 min. Finally, 50 µL of 1 M sodium hydroxide solution and 27.5 µL of distilled water were added and incubated at room temperature for 5 min. Absorbance was measured at 510 nm against a water blank reference and reported as mg of quercetin equivalents per gram sample (mg QE/g sample).

### 4.6. Tentative Phytochemical Analysis

#### 4.6.1. Extraction of Plant Samples for the Determination of Anti-Inflammatory Activity and LC-QTOF/MS

Fresh leaves were boiled using 2 g of *G. inodorum* in 100 mL of distilled water at 80 °C for 15 min and then were allowed to cool and filtered with filter paper No. 1.

#### 4.6.2. Anti-Inflammatory Analysis

Human red blood cell (HRBC) membrane stabilization was estimated by the Chippada method [27]. Separately, plant sample solutions were prepared at concentrations of 1000, 500 and 100 µg/mL of phosphate-buffered solution at pH 7.4. A red blood cell sample was added to the Alsever solution and mixed and centrifuged at 3000 rpm for 20 min at 4 °C, and only the lower red blood cell phase was collected. After that, NaCl was added and centrifuged again at 3000 rpm for 20 min at 4 °C, and the lowest phase of red blood cells was collected for analysis. Prior to analysis, 10% v/v HRBC was prepared by adding 9 mL of NaCl to 1 mL of red blood cell solution. Then, 0.5 mL of 10% v/v HRBC solution was added to a test tube with 1 mL of pH 7.4 phosphate-buffered solution and 2 mL of sodium chloride. Finally, the solution was incubated for 30 min at 37 °C and centrifuged at 3000 rpm for 10 min. The absorbance was measured at 560 nm. The percentages of protection and hemolysis were calculated using Equations (4) and (5), respectively.

Calculation method:

% Protection = 100 − [(A1/A0) × 100] (4)

where A0 = absorbance of control and A1 = absorbance of sample.

% Hemolysis = [(A1/A0) × 100] (5)

where A0 = absorbance of control and A1 = absorbance of sample.

#### 4.6.3. Analysis of Phytochemicals in *G. inodorum* via LC-QTOF/MS

The qualitative dataset of *G. inodorum* was generated by modifying the Chiwat method [28]. Briefly, the extracted samples were dissolved in methanol and cleaned with dispersive C18 SPE. Then, the solutions were passed through 0.22 μm filters before analysis. The Agilent 1290 Infinity II series coupled to a 6546 LC-QTOF/MS instrument (Agilent Tech) was used for analysis under the following conditions: LC conditions, ZORBAX Eclipse Plus C18 column (2.1 × 150 mm, 1.8 µm), 330 nm detector; 0.2 mL/min flow rate; injection volume of 10 μL; and a gradient mobile system starting with 5% acetonitrile and 95% water (1% formic acid), decreasing to 20% acetonitrile in 5 min, 30% acetonitrile in 5 min, 35% acetonitrile in 5 min, 45% acetonitrile in 5 min, 75% acetonitrile in 5 min and 95% acetonitrile until the process was completed. The chromatographic separation was accomplished using MS conditions involving an electrospray ionization (ESI) probe in positive mode, 20 psi nebulizer, 7 L/min of N_2_ flow, a 300 °C capillary temperature, 8 μL/min flow rate, 50–1000 *m*/*z* range, 4500 V capillary voltage and 280 °C dry heater. The chemical compounds were determined using the Medline library.

## 5. Conclusions

In this study, the highest gymnemic acid contents were found in air-dried and roasted 1-day-fermented leaves compared to other extracts of *G. inodorum* samples. The *G. inodorum* samples also showed antioxidant capacity. Our results suggest that methanol extract provides the highest phenolic content, while ethanol extract provides the highest flavonoid content. The anti-inflammatory results showed that the greatest protective effect was obtained with fresh leaves of *G. inodorum*. Therefore, in this study, *G. inodorum* was found to contain high phytochemical potential and can be further studied for other pharmaceutical properties.

## Figures and Tables

**Table 1:** The gymnemic acid contents of *G. inodorum* (Lour.) Decne.

Samples	Gymnemic Acid (µg/mg)
Fresh leaves	0.0194 ± 0.0001
Baked leaves	0.1020 ± 0.0122
Baked and roasted leaves	0.0786 ± 0.0086
Air-dried leaves	0.0642 ± 0.0064
Air-dried and roasted leaves	0.0974 ± 0.0115
Air-dried and roasted 1-day-fermented leaves	0.1258 ± 0.0157
Air-dried and roasted 30 s-boiled leaves	0.0578 ± 0.0066
Air-dried and roasted 60 s-boiled leaves	0.0460 ± 0.0037
*p*-value	<0.001

Each value represents the mean ± SD (*n* = 2).

**Table 2:** Antioxidant capacity of *G. inodorum* (Lour.) Decne.

Samples	DPPH (mg/mL)		ABTS (mg/mL)		FRAP (mM Fe^2+^/g Sample)
	% Inhibition	IC_50_	% Inhibition	IC_50_	
Fresh leaves	36.16 ± 7.80	36.57	69.60 ± 2.11	12.79	0.0013 ± 0.002
Baked leaves	62.58 ± 3.43	8.99	70.93 ± 2.11	1.11	0.0085 ± 0.008
Baked and roasted leaves	57.65 ± 6.42	12.59	75.58 ± 0.23	1.05	0.0070 ± 0.008
Air-dried leaves	59.80 ± 2.49	14.39	73.59 ± 4.46	1.30	0.0075 ± 0.008
Air-dried and roasted leaves	47.12 ± 12.34	14.05	ND *	ND *	0.0059 ± 0.007
Air-dried and roasted 1-day-fermented leaves	49.82 ± 11.40	12.13	ND *	ND *	0.0070 ± 0.007
Air-dried and roasted 30 s-boiled leaves	71.94 ± 3.49	9.87	73.59 ± 3.99	5.03	0.0064 ± 0.007
Air-dried and roasted 60 s-boiled leaves	69.94 ± 2.10	13.65	74.25 ± 0.23	4.05	0.0055 ± 0.006
*p*-value	0.014		>0.05		>0.05

Each value represents the mean ± SD (*n* = 2). * ND: Not detectable.

**Table 3:** The total phenolic and flavonoid contents of *G. inodorum* (Lour.) Decne.

Total Phenolic Content (µg GAE/g)			Total Flavonoid Content (µg QE/g)		
Ethanol	Methanol	H₂O	Ethanol	Methanol	H₂O
0.19 ± 0.0004	0.21 ± 0.0010	0.10 ± 0.0008	74.56 ± 28.00	71.88 ± 16.11	10.74 ± 3.63

Each value represents the mean ± SD (*n* = 3).

**Table 4:** Anti-inflammatory test results for *G. inodorum* (Lour.) Decne.

Sample	Concentration (µg/mL)	
	1000	
	% Protection	% Hemolysis
Fresh leaves	82.79 ± 2.28	17.21± 2.28

Each value represents the mean ± SD (*n* = 3).

**Table 5:** Phytochemical compounds in *G. inodorum* leaf methanol extracted by LC-QTOF/MS.

No.	Tentative Metabolite Name	Structure	RT	Matching Score (%)	*m*/*z*	Mass	Mass Diff.(db/ppm)
1	Phenethylamine	C_8_ H_11_ N	0.882	98.46	122.10	121.09	1.17
2	Kaempferol 7-O-glucoside	C_21_ H_20_ O_11_	0.992	94.47	449.11	448.10	0.83
3	6-Hydroxykaempferol 7-rutinoside	C_27_ H_30_ O_16_	0.997	99.41	633.14	610.15	0.24
4	Isoorientin2″-[feruloyl-(->6)-glucoside]	C_37_ H_38_ O_19_	1.007	95.45	809.19	786.20	0.82
5	Adenosine	C_10_ H_13_ N_5_ O_4_	1.019	98.74	268.10	267.10	1.42
6	Gymnemic acid I	C_43_ H_66_ O_14_	1.112	90.05	806.45	806.45	1.73
7	Saikosaponin L	C_42_ H_68_ O_14_	1.226	95.78	819.45	796.46	0.73
8	Momordin Ia	C_42_ H_66_ O_13_	1.228	95.81	779.46	778.45	0.06
9	Ascorbyl stearate	C_24_ H_42_ O_7_	2.478	99.15	465.28	442.29	0.46

## Data Availability

Not applicable.
